# Computer-based simulation training in emergency medicine designed in the light of malpractice cases

**DOI:** 10.1186/1472-6920-14-155

**Published:** 2014-07-27

**Authors:** Akan Karakuş, Latif Duran, Yücel Yavuz, Levent Altintop, Fatih Çalişkan

**Affiliations:** 1 Department of Medical Education, Ondokuz Mayis University Faculty of Medicine, Kurupelit, Samsun, Turkey; 2 Department of Emergency Medicine, Ondokuz Mayis University Faculty of Medicine, Kurupelit, Samsun, Turkey; 3 Department of Internal Medicine, Ondokuz Mayis University Faculty of Medicine, Kurupelit, Samsun, Turkey

**Keywords:** Computer-based simulation, Medical education, Emergency medicine, Forensic medicine

## Abstract

**Background:**

Using computer-based simulation systems in medical education is becoming more and more common. Although the benefits of practicing with these systems in medical education have been demonstrated, advantages of using computer-based simulation in emergency medicine education are less validated. The aim of the present study was to assess the success rates of final year medical students in doing emergency medical treatment and evaluating the effectiveness of computer-based simulation training in improving final year medical students’ knowledge.

**Methods:**

Twenty four Students trained with computer-based simulation and completed at least 4 hours of simulation-based education between the dates Feb 1, 2010 - May 1, 2010. Also a control group (traditionally trained, n =24) was chosen. After the end of training, students completed an examination about 5 randomized medical simulation cases.

**Results:**

In 5 cases, an average of 3.9 correct medical approaches carried out by computer-based simulation trained students, an average of 2.8 correct medical approaches carried out by traditionally trained group (t = 3.90, p < 0.005). We found that the success of students trained with simulation training in cases which required complicated medical approach, was statistically higher than the ones who didn’t take simulation training (p ≤ 0.05).

**Conclusions:**

Computer-based simulation training would be significantly effective in learning of medical treatment algorithms. We thought that these programs can improve the success rate of students especially in doing adequate medical approach to complex emergency cases.

## Background

Computer-based simulation systems are preferred for many reasons such as reducing training costs, unlimitedly repetition of the same training, evaluating the training results in real time, reducing the risk of patient harm, gaining appropriate response behavior in approaching different medical cases encountered in a hospital clinic [1-4]. Simple simulation systems (models for resuscitation and intravenous line insertion) for medical training purposes have been in use for many years in the world [3]. The priority in designing of modern medical education systems is to maximize the safety of patients and minimize the risks. In this respect, it is suggested that providing better training capabilities to physicians and medical personnel that are responsible for medical care and treatment of patients makes a major contribution to this basic principle [3]. The training programs using computerized simulation systems in radiology education which highlights visual assessment is found to be successful [5-9]. It has been shown that the use of simulation training has promising capability to gain experience in the field of pathology [10]. In the light of the data obtained from real-life experiences, it has been concluded that a computer-based interactive resuscitation system notably made better resident performance in managing pediatric emergencies in the radiology department. [11]. Medical simulation training is not only used in the branches of medicine which visual evaluations are important, but also used in clinical and surgical branches. It has been announced that a training study which simulates 15 emergency cases in different level of complexity consisted of specific learning objectives is successful within the framework of emergency medicine training program of the University of New Mexico [12]. It has been also emphasized that the health workers trained in intensive care have to check their education from point of evidence-based medicine, communication, evaluation and simulation [13]. Other advantages of simulation training are to increase the clinical experiences in a safe environment and also to provide comprehensive simulated environments which allow a route from practicing isolated tasks to more complex clinical situations [14]. It has been demonstrated that a sense of self-confidence and decision-making process develops in a person receiving the simulation training because of the tolerance to errors during training process unlike real-life [15,16]. Minimizing errors in medical education was not a new concept, but it became important for controlling and criticizing the treatment process and to solve problems after identifying the sources of failures in medicine [15]. For minimizing errors in medicine, the use of simulation training systems becomes increasingly widespread in surgical branches in developed countries [17,18]. Especially, increasing public awareness regarding to medical malpractice, the realization of virtual training applications appears to be a necessity for prevention of these errors. In this descriptive study, we aimed to evaluate the success rate of final year medical students in treating emergencies and the effectiveness of computer-based simulation training in emergency medicine education at a university hospital.

## Methods

In a university hospital, between the dates Feb 1, 2010 - May 1, 2010, a computer-based training program which simulated the common malpractice cases occurred in emergency clinics represented to final year medical students. In determined time, success rate of students trained with simulation training in doing accurate diagnosis and treatment of emergency cases was evaluated. As a control group, only traditionally trained students were chosen. Primarily, organic phosphate poisoning, gastric perforation, deep vein thrombosis and its complications, stab wound injury, drug allergy, aortic dissection, drug interactions and similar cases were simulated with computer-based simulation system in this descriptive study. These medical issues were all presented to students during their rotations with traditional training in 4th and 5th classes in both study groups. At the end of the study, students trained with computer-based simulation technique in addition to traditional education [n = 24] and students only trained with traditional education techniques [n = 24] were compared. We investigated whether both groups were applied the accurate diagnosis and treatment approaches to present cases. The accuracy of treatment approaches were evaluated by faculty members of the emergency medicine and forensic medicine department. All data obtained from the study was evaluated with Independent Samples T- test, Fisher’s Exact test and Pearson X^2^ Test.

## Results

The total number of students in the present study was forty eight. 52.1% of them (n = 25) were females and 43.8% of them (n = 21) were males. Two (4.2%) students did not reply to this question.

It was found that the accurate diagnosis and treatment approach was performed in 3.9 of 5 cases by the students trained with computer-based simulation and in 2.8 of 5 cases by students trained traditionally (t = 3.90, p ≤ 0.005) (Independent Samples T- test was used for the equality of average values].

We determined that using simulation training technique significantly increased the success rates of students especially in cases which required multi-step diagnostic and therapeutic approach (case 1 and 4) (Table 1).

**Table 1:** Comparison of rates of performing an accurate medical approach to deferent emergency cases between the students train with computer base simulation training and traditional trained

**Cases**	**Simulation training**	**Traditional trained**	**OR**	**CL**	**p**
	**n (%) success**	**n (%) success**	**(Odds ratio)**	**(%95)**	
Case 1	15[62.5%]	7[29 2%]	4.05	1.21-13.54	0.020
Case 2	24[100%]	20[83.3%]	-	-	0.109
Case 3	20[83.3%]	12[50%]	5.0	1.31-19.07	0.030
Case 4	13[54.2%]	9[37.5%]	1.19	0.62-6.24	0.247
Case 5	24[100%]	l8[75%]	2.33	0.51-10.69	0.461

In organic phosphate insecticide poisoning (case 1), we considered that the student was successful, if he/she decided the diagnosis by evaluating medical history and provided security of the scene, removal of clothing, skin decontamination, respiratory support, and giving adequate antidotes. It was found that students trained with simulation based training were significantly successful in the diagnosis and treatment of organic phosphate insecticide poisoning (Table 1). The success rate for students trained with simulation based training was 62.5% and the success rate was 29.2% for the traditionally trained students (p = 0.020).

In making accurate diagnosis and treatment approach to gastric ulcer disease and its complications like perforation of the stomach or duodenal walls (case 2), the success rate of simulation-trained students was higher than traditionally trained students (100% vs 83.3%, p = 0.109). we considered that the student was successful, if he/she decided gastric perforation diagnose in an old female patient (using non-steroid anti-inflammatory drugs for her osteoarthritis disease) by evaluating medical history and observing presence of gas under the right diaphragm in chest radiograph.

In stab wound injury (case 3), we wanted the students to recognize great vessel injury, be able to examine the pulses of lower extremities and decide the adequate radiological test as Doppler ultrasonography for being successful in the pre and post test. In applying appropriate approach for diagnosis and treatment of a stab wound injury and potential complications including great vessel injury in femoral region, the success rate for students trained with simulation based training was 83.3% (n = 20) higher than traditionally trained students (50%; n = 12; p = 0.030).

In deep vein thrombosis case (case 4), we considered that the student was successful, if he/she properly evaluated the medical history (In present simulation case an obese patient had rested for a month in her house and then attended emergency service with weakness and breath difficulty complaints). They also had to decide the diagnosis by requiring computed tomography (CT) of the chest for the diagnosis of pulmonary embolism and Doppler ultrasonography of the lower extremities for deep vein thrombosis. Simulation-trained students were more successful compared with traditionally trained students (54.2% vs. 37.5%, p = 0.247) in the performance of an accurate diagnosis and treatment of deep vein thrombosis and its complications.

In drug allergy case (case 5), we considered the students as successful, if they adequately evaluated the medical history (In present simulation case, a pediatric patient had complaints such as redness in her neck after taking an antibiotic drug prescribed by family physician), decide the diagnosis, be able to stop administration of the antibiotic treatment and initiated antihistaminic drug therapy in emergency service. The students trained with simulation based training were more successful in performing adequate therapeutic approach to a drug allergy case% 87.5 (n = 21). The success rate for traditionally trained students was 75% (n = 18) (p = 0.461).

## Discussion

Computer-based simulation has various advantages compared to traditional education and increasing number of scientific articles published dealing with the effectiveness of simulation training in literature. Mason et al. emphasized that web-based training could allow learners to properly assess a specific symptom, recognize adequate assessment techniques and assure the proper medical treatment [19]. In a previous study, Pusic et al. highlighted that in practicing of radiography interpretation, learning did not stop even after 234 cases [20]. In summary, simulation systems help learners to easily and continually practice different medical cases via internet.

In present study, students trained with computer-based training were significantly more successful than traditionally trained students in the diagnosis and treatment of organic phosphate insecticide poisoning (case 1) which requires multi-step therapeutic approach (62.5% vs. 29.2%). In Turkey, pesticide poisoning is an important public health problem [21,22]. In pesticide poisoning, removing all contaminated clothing and washing the exposed area with generous amounts of water and soap, supporting the airway and medical treatment are fundamental steps in doing adequate medical approach [23]. During applying medical treatment to pesticide poisoning, skipping of one of these steps may cause a patient to lose his/her life. The important thing was that all the students in each group had successfully passed their classes since becoming final year medical student. However, in the light of present study, we observed that a student who was successful in multiple choice test exams, which applied widely in medical education in Turkey, might not sufficient enough for doing adequate multi-step therapeutic approach to a patient. We believe that practical skills training with specialist teachers during graduate medical education is necessary for mostly seen emergencies. We reckon that computer-based simulation training will be a good choice for applying these training activities.

The overall adequate medical approach rate in approaching a stab wound injury was very low in traditionally trained students. The most seen failure done by students in doing adequate approach to a stab wound injury case was “not recognizing a great vessel injury”. In medicine knowing and performing the standard diagnose and treatment algorithm to a patient is mandatory and life saving. An inappropriate medical approach can result with death of a patient. To know and apply the standard medical approach is also one of the legal responsibilities of doctors. Medical malpractice cases occur if a patient injured or died because of a health care provider who fails to apply competently the standard treatment approach. In present study, we observed the effectiveness of simulation training in learning proper medical approach to a stab wound injury and its complications.

In emergency departments, working conditions are difficult. Emergency physicians have to take right decision urgently and adequately in crowded emergency services. Unfortunately, according to our forensic experiences, the medical education including traditional techniques is insufficient for providing the proper medical approach by doctors in emergency conditions. We think that characteristics of common malpractice cases should be evaluated and then presented to students by computer-based simulation systems for providing an adequate medical training in emergency department.

One limitation of the study is that repeating the presentation of the emergency cases in simulation group while computer based training activity, because computer-based simulation group completed their training together with continuing their normal curriculum. This may have been an obstacle in assessing the success rates of each groups because traditionally educated group observed emergency cases one less time than the simulation trained group prior to completing the examination test. But also this study design is given us a chance to see the preparedness level of traditional trained final year students in approaching real-life emergencies.

We think that educational activities using computer-based simulation systems would be useful for performing an effective training method for students. The training centers that use realistic models with medical simulation systems began to set up around the world [24]. Simulation trainings done with using computer-based mannequin showed superior outcomes in comfort in patient management but also long-term effects of simulation-based education had to be clarified [25].

In parallel with the advances in computer science, it is clear that a rapid development of simulation training systems is expected. So we think that in designing phase of these systems, collaboration between scientists from different branches of science as computer engineers, emergency medicine and forensic medicine specialist is needed for improving emergency medicine education.

For performing an adequate treatment approach in emergency clinics, it is important to act with current medical diagnosis and treatment guidelines. In a study consisted of 30 medical malpractice cases, researchers found that inadequate treatment approaches in 14 cases (47%), cautiousness in 10 cases (33%), neglect in 4 cases (13%) and the wrong diagnosis in 2 cases (7%) in Turkey [26]. We reckon that after evaluating commonly seen malpractice cases, training programs with computer-based simulation systems should be carried out in emergency medicine education.

In Turkey, education programs in medical schools for communication skills using standardized patients both in training and assessment have steadily increased over the past ten years [27], but also designing and using of computer-based simulation programs in medical education has not commonly occurred yet. In the light of present study, development of a computer-based simulation system completed in December of 2011 at Medical Faculty of Ondokuz Mayıs University with the computer-engineering department. By this system, researchers presented common malpractice cases to the students and declared that the preparedness level of final year medical students for an adequate approach to simulated emergency cases increased [28].

## Conclusion

Computer-based simulation training designed in the light of forensic medicine experiences seems to increase the success rates of students in complex emergency cases. Although the success rate of simulation-trained group was higher than traditionally educated group, the overall success rate remains not very high in simulation-trained group. We considered that each failed medical approach in simulation training is equal to death of a patient in real life and full success achieved only in one case in simulation group. In the light of this finding, we did not consider that computer-based simulation training is not effective in medical education. Because, during the educational activities, physiological factors such as “not to show enough interest”, “to forget the information which is not being practiced or not being repeated” may have considered as major obstacles in front of a complete success. We proposed simulation training as a solution to this problem. More importantly, we thought that internet accessible web-based simulation training can easily repeatable, so by repeating the simulation training in complex medical cases could better improve student success.

## Competing interests

The authors have no relevant conflicts of interest to declare. No funding was received for the present study.

## Authors’ contributions

AK designed and implemented the computer-based learning course under the guidance of LA. AK wrote the manuscript with edits provided by LD, YY, and FÇ. All authors read and approved the final manuscript.

## Pre-publication history

The pre-publication history for this paper can be accessed here:

http://www.biomedcentral.com/1472-6920/14/155/prepub
